# Salt Stress Induces Contrasting Physiological and Biochemical Effects on Four Elite Date Palm Cultivars (*Phoenix dactylifera* L.) from Southeast Morocco

**DOI:** 10.3390/plants13020186

**Published:** 2024-01-10

**Authors:** Ibtissame Benaceur, Reda Meziani, Jamal El Fadile, Jan Hoinkis, Edgardo Canas Kurz, Ulrich Hellriegel, Fatima Jaiti

**Affiliations:** 1 Biodiversity, Environment and Plant Protection Team, Faculty of Sciences and Technology, My Ismail University, Errachidia 52000, Morocco; 2 National Institute for Agronomic Research, CRRA, Meknes 50000, Morocco; 3 National Institute for Agronomic Research, CRRA, Errachidia 10090, Morocco; 4 Center of Applied Research, Karlsruhe University of Applied Sciences, Moltkestr. 30, 76133 Karlsruhe, Germany

**Keywords:** salt stress, date palm (*Phoenix dactylifera* L.), salinity tolerance, Mejhoul, Boufeggous, Tafilalet

## Abstract

Understanding the response of date palm (*Phoenix dactylifera* L.) cultivars to salt stress is essential for the sustainable management of phoeniculture in Tafilalet, Morocco. It offers a promising avenue for addressing the challenges presented by the increasing salinity of irrigation waters, especially because farmers in these regions often lack the necessary knowledge and resources to make informed decisions regarding cultivar selection. This study addresses this issue by investigating the performance of the most relied on cultivars by farmers in Tafilalet, namely Mejhoul, Boufeggous, Nejda, and Bouskri. These cultivars were exposed to a sodium chloride treatment of 154 mM, and their performances were evaluated over a three-month period. We examined the growth rate, photosynthesis-related parameters, pigments, water status in plants, and biochemical compounds associated with oxidative stress, osmotic stress, and ionic stress. Principle component analysis (PCA) effectively categorized the cultivars into two distinct groups: salt-sensitive (Mejhoul and Nejda) and salt-tolerant (Boufeggous and Bouskri). These findings provide valuable insights for farmers, highlighting the advantages of cultivating Boufeggous and Bouskri cultivars due to their superior adaptation to salt conditions. These cultivars exhibited moderate decrease in shoot growth (25%), enhanced catalase activity, a smaller increase in anthocyanin content, and greater enhancement in organic osmolytes compared with salt-sensitive cultivars like Mejhoul (experiencing an 87% reduction in shoot elongation) and Nejda (exhibiting the highest reduction in leaf area). Furthermore, the Na^+^/K^+^ ratio was positively influenced by salt stress, with Mejhoul and Nejda recording the highest values, suggesting its potential as an indicator of salt stress sensitivity in date palms.

## 1. Introduction

Soil salinity is a critical constraint that significantly hinders agricultural productivity globally and within the context of Morocco [1]. Several research findings indicate that soil salinity affects a significant proportion, estimated to range between 40% and 45%, of arable land worldwide, leading to substantial economic losses [2,3]. Morocco exemplifies the challenges posed by soil salinization, as recent studies indicate that over 5% of the country’s soils are already impacted to varying extents. This has led to decreased crop yields and compromised quality, exerting a detrimental impact on the agricultural sector [4]. Furthermore, the excessive adoption of saline water irrigation practices is anticipated to contribute to an increase in reported saline regions in the future, a phenomenon that is expected to be particularly prevalent in arid and semiarid regions in which the rate of evapotranspiration surpasses that of precipitation [5,6]. The Tafilalet plain in Morocco is an exemplary hyper-arid to desertic region with a highly valuable ecosystem characterized by significant ecological and natural resources, including fertile agricultural soil and essential groundwater [7]. However, the region is also experiencing rapid changes resulting from both natural and human-induced factors, with a primary concern being soil salinity. The cultivation of the date palm (*Phoenix dactylifera* L.), which is considered one of the most important fruit species in this region, is primarily driven by its remarkable productivity, nutritional quality, ability to preserve oasis ecosystems, and capacity to create a microclimate that is conducive to agriculture in arid conditions. As a result, it offers significant income opportunities for the local population, with estimates suggesting that it contributes to up to 20% to 60% of the overall income of the local farmer community, thereby enabling the subsistence of numerous families whose livelihoods depend directly and indirectly on the products derived from this fruit tree [7]. In general, the production in these areas is distributed as follows: 30% for self-consumption, 20% for animal feed, and 50% for commercialization [8].

According to the Moroccan Agency for Agricultural Development [9], the phoenicultural heritage of Morocco encompasses 7.2 million plants, representing approximately 5.1% of the total world population. The provinces of Ouarzazate (Drâa Valley), Errachidia (Tafilalet and Ziz valleys), and Tata (Bani) are responsible for almost 90% of the national population. From a genodiversity perspective, the Moroccan phoenicultural heritage is highly diverse, comprising nearly 453 different genotypes, consisting of 52.3% cultivars and 47.7% of genetic resources from seedlings [7]. The representativeness of each cultivar varies from one palm grove to another, with the Mejhoul cultivar being dominant only in the palm groves of Tafilalet and Ziz, while the Boufeggous cultivar is encountered in almost all Moroccan palm groves. The Ziz-Tafilalet, Drâa, and Bani palm groves are the most genetically diverse and feature 12 cultivars that are the most valued by farmers, namely Mejhoul (with only 0.3% of the varietal mix), Boufeggous, Jihel, Bouskri, Bousthammi Noir, Bouslikhène, Outokdime, Bouittob, Ahardane, Aguelid, Taabdount, and Aziza Bouzid [7].

Date palms exhibit remarkable resilience in harsh environmental conditions, including hot and arid terrains [10]. However, date palm species continue to face significant threats from abiotic stresses, which negatively impacts various aspects of the plant’s physiology, including leaf size, stem extension, root proliferation, and water–plant interactions [11]. The complex salt stress tolerance mechanisms in plants involve a combination of intricate physiological, biochemical, and molecular responses. Recent research efforts have been directed toward comprehending these mechanisms, with the goal of enhancing salt stress tolerance in crops. Some mechanisms that have been used include ion exclusion, osmotic adjustment, antioxidant defense, and hormonal regulation. Ion exclusion involves the selective uptake and compartmentalization of indispensable ions while excluding toxic ions like sodium from sensitive tissues [12]. Osmotic adjustment helps maintain cellular water potential and restrict water loss under salt stress conditions [13]. Antioxidant defense systems, including enzymes such as superoxide dismutase and catalase, aid in scavenging reactive oxygen species generated during salt stress [14]. Hormonal regulation, particularly abscisic acid, plays a crucial role in coordinating plant responses to salt stress [15]. Genetic variations and differences in gene expression related to these tolerance mechanisms result in cultivar-specific responses to salt stress, even among cultivars of the same species. In the date palm species (*Phoenix dactylifera* L.), significant research has highlighted various adaptive mechanisms. These mechanisms include the substantial impact on the regulation of a diverse range of genes associated with abiotic stress. At the biochemical level, the closure of stomata results in a limited assimilation of CO_2_ by leaves, triggering the activation of the photorespiratory pathway. This process leads to oxidative damage and the excessive production of reactive oxygen species (ROS). As a response, plants employ adaptive strategies such as minimizing transpirational losses and transitioning to smaller leaves, effectively reducing surface area while enhancing leaf thickness [16]. Furthermore, an accumulation of osmolytes, including total soluble sugars, proteins, proline, and glycine betaine, has been observed in plants. These osmolytes play a crucial role in maintaining vital cellular functions [17]. Additionally, plants produce key enzymatic and nonenzymatic antioxidant molecules that play a vital role in detoxifying ROS. The involvement of plant hormones is also evident in these adaptive mechanisms [18]. In the recent literature, studies such as [19] provide valuable insights into these mechanisms and cultivar-specific responses to salt stress in date palms.

The main objectives of this study are twofold. Firstly, it aims to assess the tolerance of high fruit quality genotypes in Morocco toward salt stress, with the intention of assisting farming communities in the selection of cultivars that are well-suited to flourish under such conditions. Secondly, this study aims to identify the key distinguishing traits of tolerance or susceptibility exhibited by each individual cultivar or group of cultivars that may be used in future studies to screen other date palm cultivars within or outside Morocco. Consequently, this study aims to address two primary questions: Firstly, do the selected Moroccan date palm cultivars exhibit uniform responses to salt stress? Secondly, if not, which cultivars demonstrate tolerance and sensitivity among the four selected, and what are the critical parameters significantly affected by salt stress that could be utilized to screen other varieties within or outside Morocco?

## 2. Materials and Methods

### 2.1. Plant Materials, Plant Culture, and Salinity Stress

The Moroccan National Institute for Agronomic Research (National Laboratory of Date Palm Tissue Culture; National Institute for Research, CRRA-Errachidia, Rabat, Morocco) provided four cultivars for this study, namely Mejhoul, Boufeggous, Nejda, and Bouskri. These cultivars were selected to perform the screening for salt stress tolerance based on their exemplary fruit quality [20]. Table 1 presents a comprehensive inventory and a concise overview of these genotypes’ characteristics.

The growth medium used for growing the plants in this study consisted of a blend of sand and peat in a volume ratio of 2:1. Prior to the application of salt treatment, the chemical composition of this mixture can be described as follows: The pH of the growth medium was measured at 7.2. The electrical conductivity (EC) was determined to be 0.8 ms.cm^−1^. Additionally, the substrate’s salinity was found to be 0.51 g.L^−1^, indicating a relatively low salt content.

Prior to the formal experiments, preliminary trials involving different NaCl concentrations (52 mM, 102 mM, and 154 mM) at different date palm growth stages were conducted to determine the appropriate salt level that elicits significant effects on the plants. The sole NaCl concentration that demonstrated statistically significant variances within a 3-month timeframe among the tested concentrations was determined to be 154 mM. Consequently, this particular level was selected as the appropriate concentration for the subsequent study. It is worth noting that the parameters utilized in this preliminary investigation were the same as those employed in the current study. These parameters encompassed various phenotypical (such as plant height, leaf area, number of intact leaves, number of chlorosed leaves, number of neoformed leaves), physiological (including CCI, stomatal conductance, chlorophyll fluorescence), and biochemical aspects (such as proline contents, total soluble sugars, antioxidant enzymes, total phenolic compounds, H_2_O_2_ concentrations).

**Figure 1:**
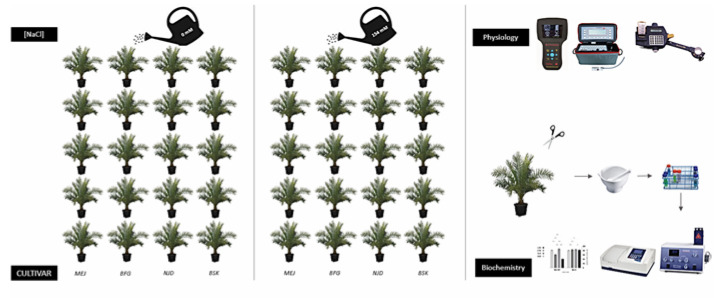
Schematic representation of the experimental design employed for screening the salt stress tolerance of four distinct cultivars of date palm (*Phoenix dactylifera* L.). The experimental design involved the utilization of two salinity levels, namely 0 mM and 154 mM NaCl, to evaluate the response of four different date palm cultivars, namely Mejhoul (MEJ), Boufeggous (BFG), Nejda (NJD), and Bouskri (BSK), to salt stress. A total of 40 plants from each cultivar were subjected to each salinity level, resulting in a total of 80 pots per cultivar. Following a period of three months, comprehensive morphophysiological and biochemical analyses were conducted to assess the impact of salt stress on the cultivars under investigation.

To represent the population of each cultivar, a total of 40 vitro plants, aged 9 years and exhibiting similar phenotypic traits (height, number of intact leaves, similar CCI levels), were selected for each treatment (0 mM and 154 mM). This selection process resulted in 40 plants for the control batch and an additional 40 plants for the stressed batch.

(Figure 1). Sodium chloride (NaCl) was incrementally introduced over a span of 15 consecutive days utilizing a series of dilutions with varying concentrations (25%: 38.5 mM, 50%: 77.1 mM, 75%: 115.6 mM, and 100%: 154 mM) in order to mitigate the occurrence of osmotic shock. Salt-treated batches were irrigated twice a week with a solution of tap water containing 154 mM NaCl, with a volume of 500 mL per application. On the other hand, control lots received tap water only. After 3 months, evaluations of both phenotypic and physiological characteristics were conducted. Leaves were then harvested, and biochemical analyses were performed (Figure 1).

### 2.2. Phenotypical Measurements

Measurements of the plant heights (cm) were taken using a tape measure placed at the collar level, with the zero-meter mark serving as the reference point, at the onset of the experimental trials, as well as after a period of three months [22]. This methodology enabled the determination of the shoot elongation rate (in centimeters). Additionally, the leaf areas (in square centimeters) were ascertained using the planimeter Ushikata X-PLAN 380 dIII (Tokyo, Japan).

### 2.3. Physiological Measurements

#### 2.3.1. Stomatal Conductance and Chlorophyll Fluorescence

The assessment of stomatal conductance was accomplished between the hours of 11:00 a.m. and 12:00 p.m., whereby the measurements were taken in millimoles per square meter per second (mmol m^−2^ s^−1^) from fully expanded leaves. This was performed through the use of an AP4 porometer, manufactured by Delta-T Devices, Cambridge, UK. Similarly, the maximum quantum yields of the photosystem II were evaluated by means of the Opti-Sciences 30P^+^ fluorimeter, with measurements being recorded 30 min post-application of the leaf clips.

#### 2.3.2. Relative Water Content and Electrolyte Leakage

Relative water contents were estimated after 90 days of treatments according to [23]. Briefly, leaves were weighed to determine the fresh weights (FW), placed in petri dishes with water, and incubated at room temperature for 24 h to measure the turgid weight (TW) before drying the samples at 80 °C for 24 h to obtain the different dry weights (DW). The relative water contents (RWC) were then calculated using the formula:

RWC (%) = ((FW − DW)/(TW − DW)) × 100

Electrolyte leakage was determined at the end of the trials using 0.1 g of fresh leaf samples that were incubated in a 100 mL deionized water bath (40 °C, 30 min). The electrical conductivity was measured using an EC meter and recorded as EC_1_. The samples were re-incubated in a water bath (100 °C, 15 min), and the electrical conductivity was measured again (EC_2_). The electrolyte leakage percentage was calculated using the following formula [24]:

% EL= (EC_1_/EC_2_) × 100

### 2.4. Biochemical Measurements

#### 2.4.1. Total Soluble Sugar Content

For the determination of total soluble sugar content, a homogenate comprising 0.1 g of leaf material and 4 mL of 80% ethanol was subjected to centrifugation at 5000 rpm for 10 min. The supernatant was collected, and the remainder was resuspended via the addition of 2 mL of ethanol and subjected to a second centrifugation. The two supernatants were then utilized to quantify the total quantity of soluble sugars using the procedure outlined by [25].

#### 2.4.2. Proline Contents

Proline contents were measured in accordance with the methodology described by [26]. Randomly selected leaf samples (0.1 g) from each treatment group were homogenized in a 5 mL aliquot of 3% (*w*/*v*) sulfosalicylic acid and filtered through filter paper. The resulting mixture was then subjected to the addition of glacial acetic acid and acidic ninhydrin, heated at 100 °C for 1 h and subsequently cooled. Extraction with toluene was performed, and the chromophore containing toluene was isolated and its optical density measured at λ = 520.

#### 2.4.3. Enzymatic Activities

The extraction was performed according to an optimized protocol described by [27]. Fresh leaves (0.5 g) were ground at 4 °C in a 5 mL solution containing 0.1 M potassium phosphate buffer (pH 7.0), 0.1 g polyvinylpolypyrrolidone (PVPP), and 0.1 mmol ethylenediaminetetraacetic acid (EDTA). The homogenate was centrifuged at 18,000× *g* and 4 °C for 10 min, and the supernatants were stored at −20 °C for further biochemical analysis.

#### 2.4.4. Activity of Catalases

The enzyme extract (50 μL) was added to 1 mL of the catalase reaction solution containing 50 mM phosphate potassium buffer (pH = 7) and 15 mM hydrogen peroxide. The degradation of H_2_O_2_ was monitored by observing the decrease in absorbance at 240 nm. The activity of catalase is quantified in units, where 1 unit of catalase converts 1 mmol of H_2_O_2_ per minute.

#### 2.4.5. Peroxidases Activity

The enzyme extract (33 μL) was mixed with 1 mL of the peroxidase reaction solution containing 13 mM guaiacol, 5 mM hydrogen peroxide, and 50 mM phosphate buffer (pH = 7). The absorbance of the sample was measured at 470 nm. POX activity was estimated in terms of enzyme units per gram of fresh matter (EU/min/g FW). An enzyme unit is defined as the quantity of enzyme required to induce a change in optical density (OD) equivalent to 0.1.

#### 2.4.6. Total Phenolic Contents

The total phenolic fraction was extracted in methanol (MeOH 80%). The recovered supernatants from the various grinds were used for the determination of polyphenols and flavonoids contents. Quantifications were performed according to the Folin–Ciocalteu reagent method. A total of 50 μL of the phenolic extract was added to test tubes containing 250 μL of the Folin’s reagent, 500 μL of sodium bicarbonates (NaHCO_3_ 20%), and 2.5 mL of distilled water. An incubation at 40 °C for 30 min was conducted and the different optical densities were read at λ = 760 nm.

#### 2.4.7. H_2_O_2_ Levels

H_2_O_2_ concentrations were determined according to the protocol of [28]. A total of 0.25 g of fresh leaves were homogenized in a cold mortar with 5 mL of 10% trichloroacetic acid (TCA). The homogenate was centrifuged at 15,000 rpm for 15 min at 4 °C, and the supernatant was collected to determine the H_2_O_2_ content after adding 0.5 mL of 10 mM phosphate buffer, pH 7, and 1 mL of 1M potassium iodate to 0.5 mL of the collected supernatant. The absorbance was read at 390 nm after 1 h of incubation in the dark.

#### 2.4.8. Total Chlorophyll and Anthocyanin Contents

A modified method outlined by [29] was used to determine the total chlorophyll (TC) content. Leaf samples (0.1 g) were soaked in 10 mL of 80% acetone solution for 3 days in the dark to extract TC. The absorbance of the TC extract was measured at 645 and 663 nm, and the TC content was calculated using the equation:

TC (mg g^−1^) = 20.2(A645) + 8.02(A663) × (V/1000)/W

where A645 and A663 represent absorbance of TC extract at 645 and 663 nm, respectively, V is the total extract volume, and W is the leaf fresh weight.

The modified procedure of [30] was used to analyze the total anthocyanin (TA) content. Approximately 0.1 g fresh weight of leaf tissues was soaked for 72 h in 10 mL of acidified ethanol (ethanol: 1 N HCl, 85:15 *v*/*v*). The suspension was filtered through Whatman No.1 filter paper, and the absorbance was measured at 535 nm.

#### 2.4.9. Ion Analysis

To obtain a homogenous representative sample for ion analysis, leaves were dried in an oven at 70 °C for 2 days. The dried samples were then ground to a fine powder. A 0.1 g portion of the powder was added to 10 mL of 0.5 M HNO_3_ and placed in an oven at 80 °C for 1 h. After 1 h, the mixture was mixed and left to cool. Sodium and potassium were estimated using flame photometry with standard solutions of both sodium and potassium.

### 2.5. Salt Tolerance Evaluation

The monitored parameters were normalized using the salt tolerance coefficient (STC), which was used to calculate the MFV. In addition, MFVs were used to rank date palm genotypes according to degree of salt tolerance. The higher the average MFV, the higher the salt tolerance [31]. Salt tolerance coefficients (STC) were calculated using the formula:

STC = (Value for the NaCl treated plant/Value for the control) × 100

MVF value was calculated using the following formula for every trait described by [31]:

MVF_1_ = (STC_i_ − STC_min_)/(STC_max_ − STC_min_)

MVF_2_ = 1 − ((STC_i_ − STC_min_)/(STC_max_ − STC_min_))

where STC_i_ is the salt tolerance coefficient of a specific genotype. STC_min_ and STC_max_ are the minimum and maximum salt tolerance coefficient value amongst all genotypes, respectively. MFV_2_ was used for the traits that are inversely related for salinity tolerance (the lower the value, the higher the tolerance to salt, for example, leaf Na^+^ content and H_2_O_2_ contents).

The MFV produces a value ranging between 0 and 1. The MFV of all traits for each genotype were averaged together and ranked the degree of salinity tolerance amongst date palm cultivars (from 1 to 4).

### 2.6. Statistical Analysis

The present study employed Origin Pro software (version 2023b) for data visualization and analysis purposes. Specifically, principal component analysis (PCA) was conducted to assess the variability in biochemical and physiological parameters across the diverse cultivars under investigation, which were subjected to designated concentrations of sodium chloride (NaCl). To determine the statistical significance of variations in both biochemical and morphophysiological metrics among the different cultivars under salt stress, the analysis of variance (ANOVA) test was employed. Subsequently, the post hoc Tukey test was utilized to conduct further examinations of the significance levels between samples. The threshold for statistical significance was established at a *p* value of less than 0.05. Notably, all statistical analyses were conducted using GraphPad Prism 10.0.0 software.

## 3. Results

### 3.1. Pheno-Physiological Evaluation

**Figure 2:**
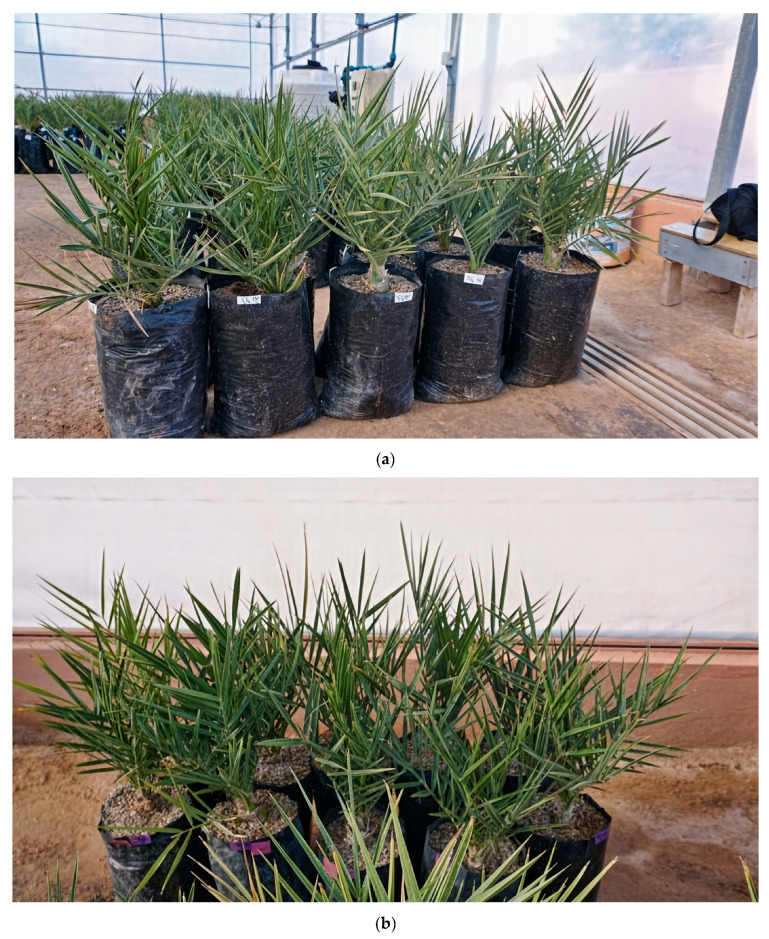

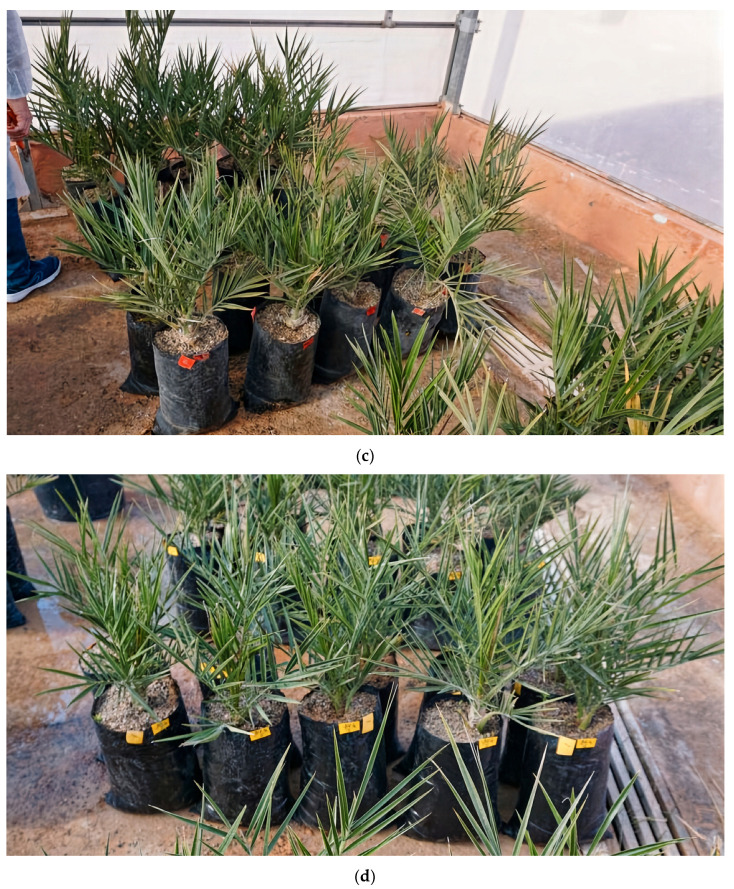
Control batches (0 mM NaCl) of the four date palms (*Phoenix dactylifera* L.) utilized in this study: (**a**) Nejda, (**b**) Boufeggous, (**c**) Mejhoul, and (**d**) Bouskri.

Figure 2 illustrates the control batches (0 mM NaCl) of the four cultivars utilized in the present study, namely Nejda, Boufeggous, Mejhoul, and Bouskri.

**Figure 3:**
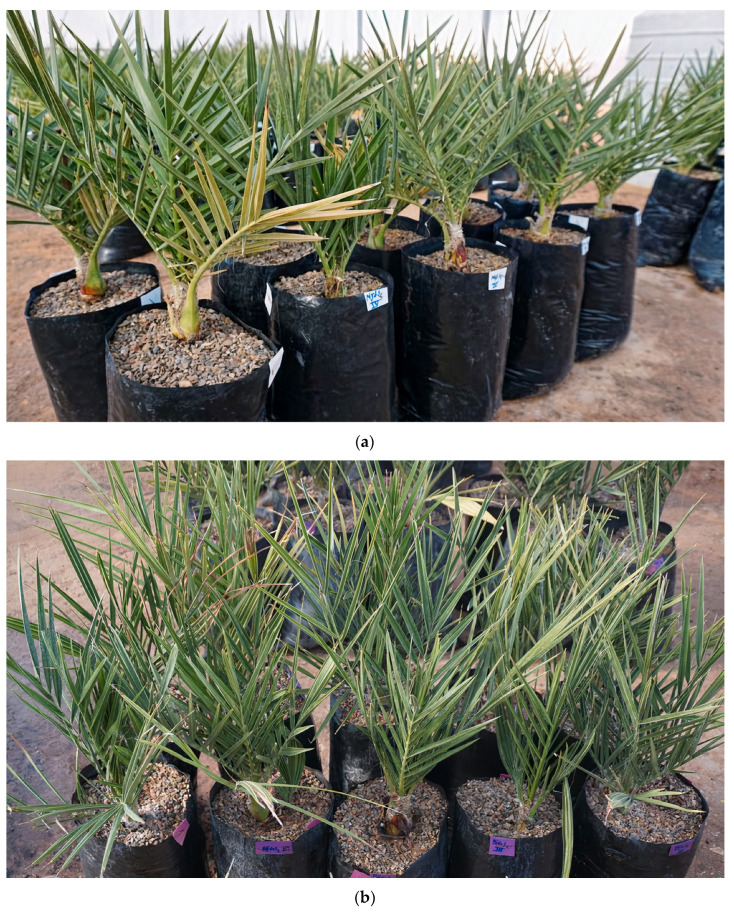

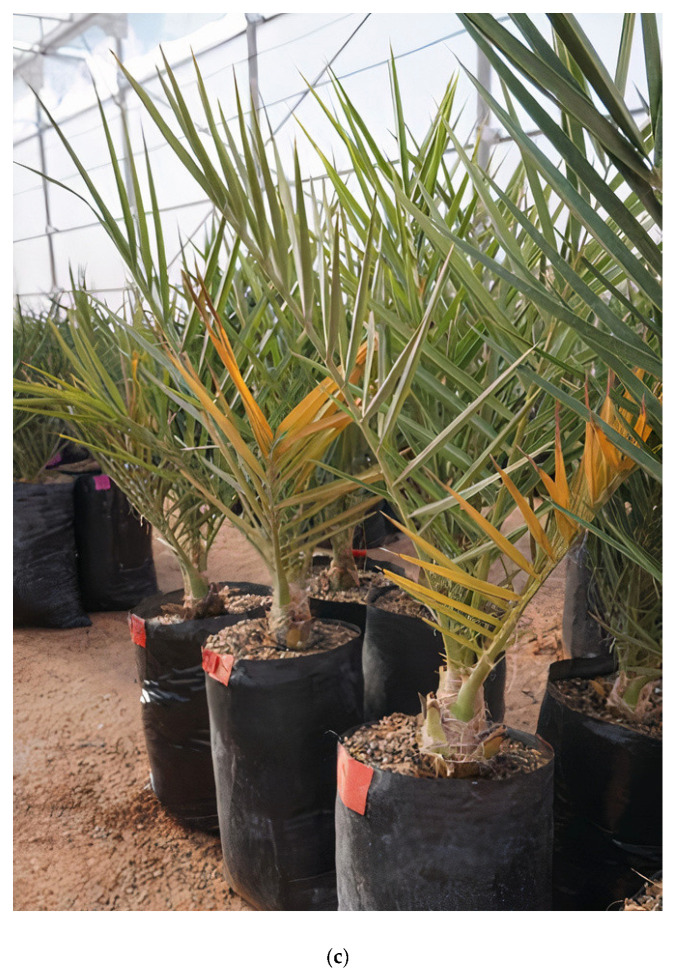

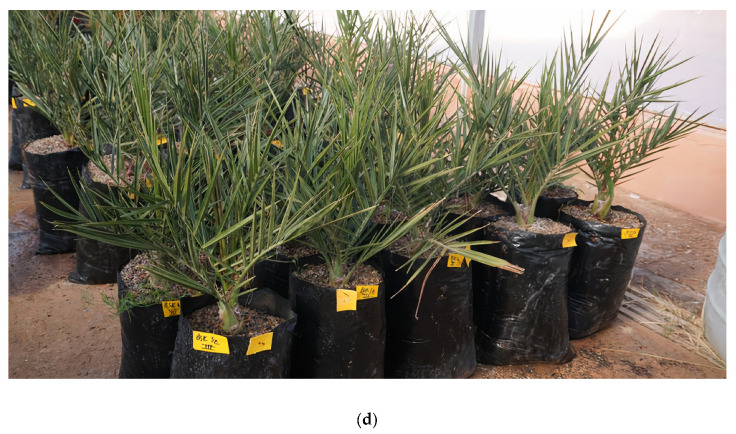
Phenotypic variations of the four date palm (*Phoenix dactylifera* L.) cultivars after three months of exposure to salt stress (154 mM NaCl): (**a**) Nejda, (**b**) Boufeggous, (**c**) Mejhoul, and (**d**) Bouskri.

After three months of exposure to salt stress (154 mM NaCl), the effects of salt stress and the variation in the phenotype of the four used cultivars are presented in Figure 3.

The salt stress conditions resulted in a reduction in the measured phenophysiological traits, as outlined in Table 2. More specifically, the Mejhoul genotype exhibited the most substantial decrease, registering an 87% reduction in shoot elongation when compared with the other genotypes. Conversely, the Boufeggous genotype displayed a relatively modest reduction of 25.09% in shoot elongation. Analysis of leaf area revealed values ranging from 22.1 cm^2^ to 32.36 cm^2^ for control plants, whereas stressed plants exhibited values ranging from 8.2 cm^2^ to 22.41 cm^2^. Reduction rates in leaf area varied between 29% and 82%, with Nejda and Boufeggous recording the aforementioned figures, respectively (as indicated in Table 2) (*p* < 0.0001).

**Figure 4:**
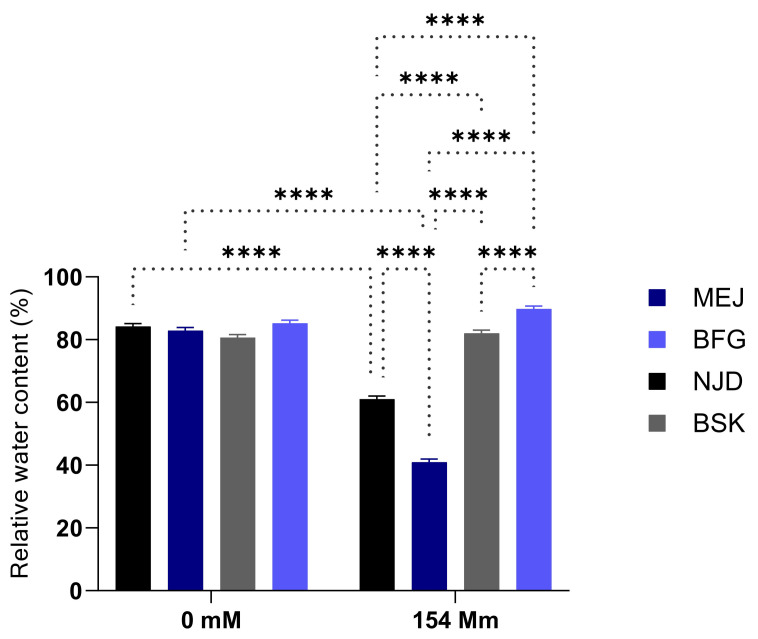
Salt stress effect on relative water contents in four Moroccan date palm (*Phoenix dactylifera* L.) cultivars. Means were separated using Tukey’s test at *p* ≤ 0.05. Bars represent mean ± SE (*n* = 40). Significant (*p* ≤ 0.05) differences are marked with asterisks. **** significant difference at *p* < 0.0001.

Additionally, a notable decrease in the relative water content (expressed as a percentage) was noted, with the exception of the Boufeggous and Bouskri genotypes, as illustrated in Figure 4. The observed differences in this response among the four cultivars were statistically significant (*p* < 0.0001) under the conditions of salt stress. Based on this significant impact, the genotypes were classified into two distinct categories. The first category consisted of two genotypes, Mejhoul and Nejda, which displayed reductions in relative water content of 50.8% and 27.47%, respectively, when compared to the control batches. In contrast, the genotypes belonging to the second category, namely Boufeggous and Bouskri, did not exhibit any significant reductions in this specific parameter.

The stomatal conductance (SC) exhibited notable variations across the four cultivars (*p* < 0.0001). The decrease in SC due to salt stress ranged from 19.35 mmol/m^2^/s to 43.32 mmol/m^2^/s (Table 2). In control plants, the range of SC values was between 67.95 mmol/m^2^/s and 86.94 mmol/m^2^/s. Notably, the Boufeggous and Bouskri genotypes displayed significantly higher stomatal conductance compared with the Nejda and Mejhoul group (*p* < 0.001). In the spectrum of genotypes examined, the most notable and least conspicuous declines in stomatal conductance were observed in Nejda and Bouskri. Notably, Nejda demonstrated the highest magnitude of reduction in stomatal conductance, amounting to 77.74%. This was followed by Mejhoul with a reduction rate of 73.19%, Boufeggous at 48.11%, and Bouskri at 36.20%.

Salt stress induced the highest levels of membrane damage in both the Mejhoul and Nejda cultivars among the four cultivars examined, as indicated in Table 2. Conversely, the Boufeggous and Bouskri cultivars exhibited significantly lower levels of electrolyte leakage, with percentages of 21.76% and 24.16%, respectively (*p* < 0.001). Indeed, when exposed to salt stress, the Mejhoul cultivar exhibited a fourfold increase in electrolyte leakage. Similarly, the Boufeggous and Bouskri cultivars demonstrated a 2-fold increase, whereas the Nejda cultivar experienced a 1.6-fold increase in electrolyte leakage.

The impact of salinity on the maximum photosynthetic efficiency of PSII, as assessed by the chlorophyll fluorescence Fv/Fm ratio, resulted in minimal reductions in the Boufeggous (6.82%) and Bouskri (4%) cultivars, with no statistically significant differences observed compared to the control condition. In contrast, the reductions in the Fv/Fm ratio were considerably more pronounced and significant for the Nejda and Mejhoul cultivars. Particularly noteworthy was the substantial decrease observed in the Nejda cultivar, amounting to 26.02%, resulting in an Fv/Fm ratio value of 0.546. Following closely, the Mejhoul cultivar exhibited a reduction of 22.97% in the Fv/Fm ratio (Table 2).

### 3.2. Biochemical Evaluation

#### 3.2.1. Na^+^/K^+^ ratio

**Figure 5:**
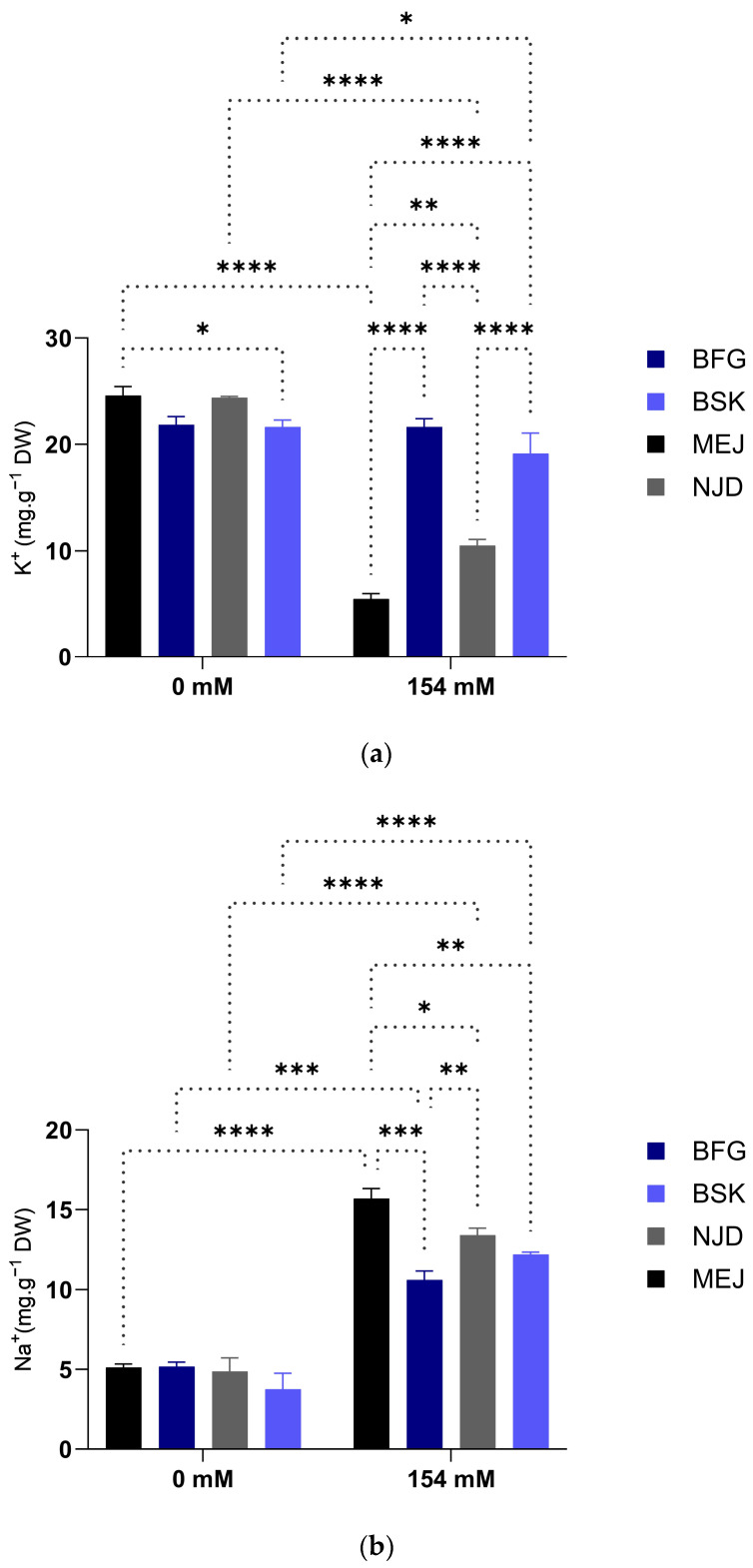

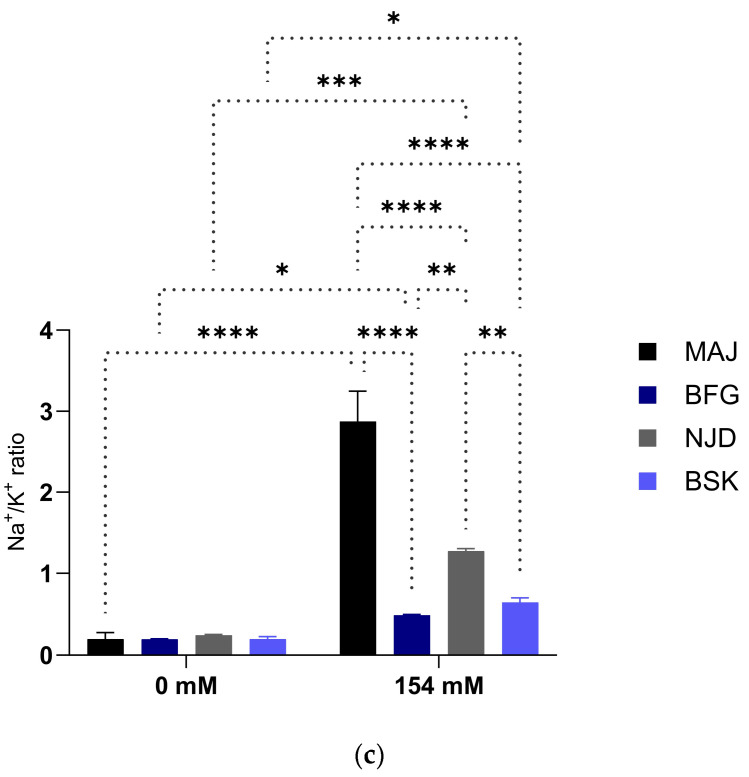
Impact of salinity treatment on Na^+^, K^+^, and Na^+^/K^+^ ratio in the leaves of four Moroccan date palm cultivars grown under normal (0 mM NaCl) and saline (154 mM NaCl) conditions. (**a**) Effect of salt treatment on K^+^ contents; (**b**) effect of salt treatment on Na^+^ contents; and (**c**) effect of salt treatment on Na^+^/K^+^ ratio. Means were separated using Tukey’s test at *p* ≤ 0.05. Bars represent mean ± SE (*n* = 3). Significant (*p* ≤ 0.05) differences are marked with asterisks. * Significant difference at *p* < 0.05; ** significant difference at *p* < 0.005; *** significant difference at *p <* 0.001; and **** significant difference at *p <* 0.0001.

The Na^+^/K^+^ ratios of the date palm vitro plants employed in this investigation exhibited an increase across all cultivars when subjected to salt stress, as illustrated in Figure 5.

Specifically, the Na^+^/K^+^ ratio in the Mejhoul cultivar experienced a dramatic increase from 0.19 to 2.87 (a 15-fold increase) under salt stress conditions. On the other hand, the Boufeggous cultivar demonstrated only a slight increase in the Na^+^/K^+^ ratio of 2.52 times (Figure 5).

#### 3.2.2. Organic Osmolytes Accumulation

**Figure 6:**
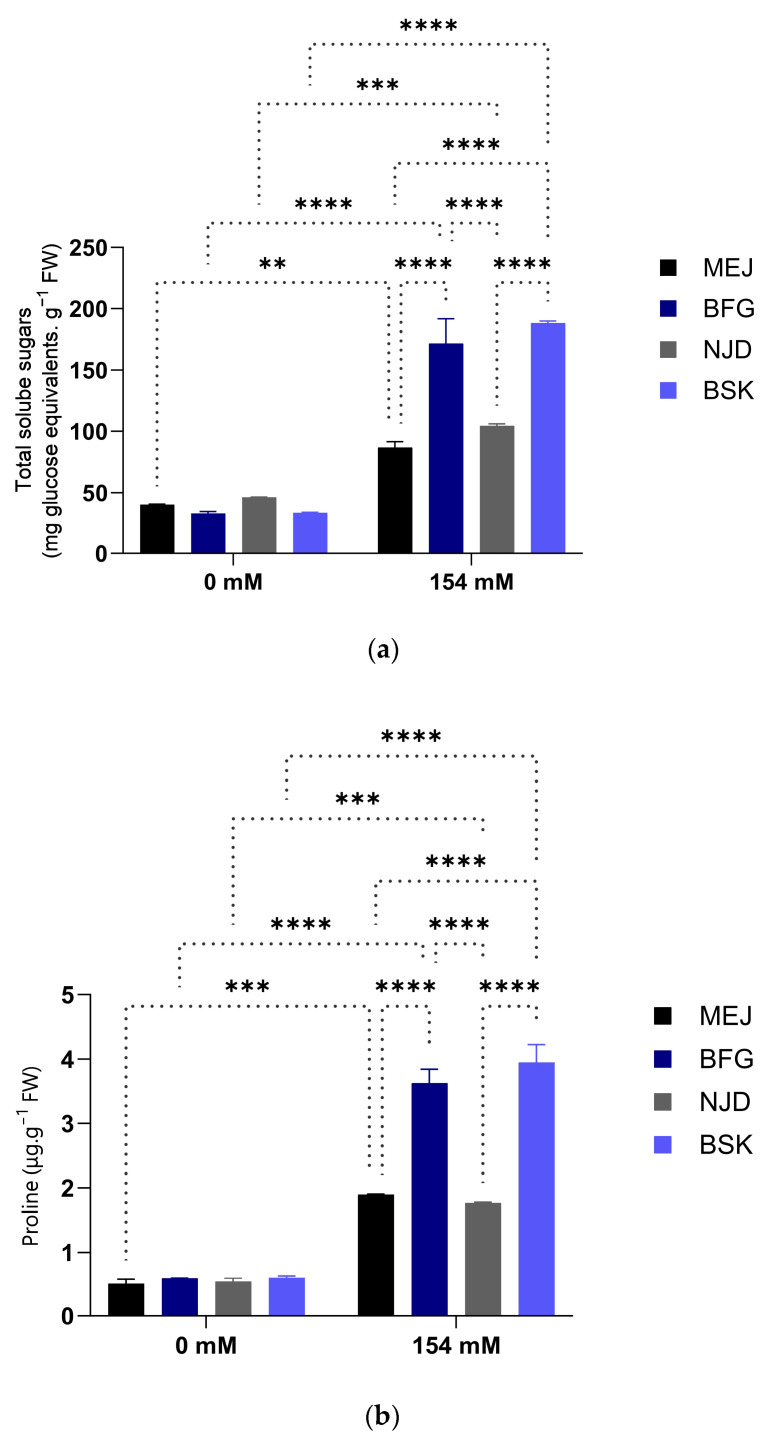
Influence of salt stress on organic osmolytes contents in leaves of four Moroccan date palm (*Phoenix dactylifera* L.) cultivars. (**a**) Effect of salt treatment on total soluble sugars contents and (**b**) effect of salt treatment on proline contents. Means were separated using Tukey’s test at *p* ≤ 0.05. Bars represent mean ± SE (*n* = 3). Significant (*p* ≤ 0.05) differences are marked with asterisks. ** significant difference at *p* < 0.005; *** significant difference at *p* < 0.001; and **** significant difference at *p* < 0.0001.

Salt stress elicited a conspicuous increase in the accumulation of proline and soluble sugars in the leaves of all four cultivars examined, as depicted in Figure 6, when compared with the control condition.

Notably, the Bouskri cultivar exhibited the highest proline accumulation, with a value of 3.94 µg.g^−1^ FW (a six-fold increase compared with the control). Conversely, the Mejhoul plants exposed to 154 mM NaCl demonstrated the lowest proline accumulation, with a three-fold concentration relative to the control (Figure 6).

#### 3.2.3. TC and TA Contents

**Figure 7:**
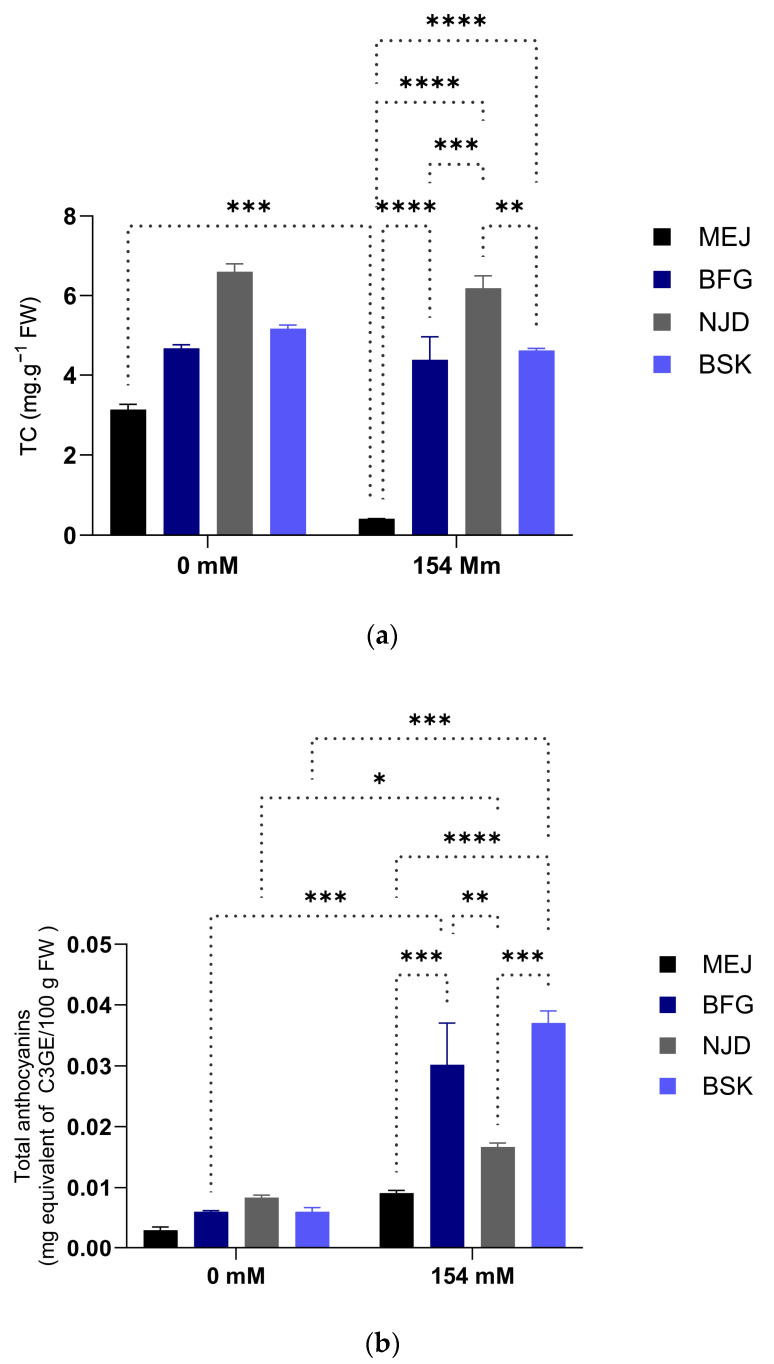
Influence of salt stress on some plant pigment contents in the leaves of four Moroccan date palm (*Phoenix dactylifera* L.) cultivars. (**a**) Effect of salt treatment on total chlorophyll contents and (**b**) effect of salt treatment on total anthocyanin contents. Means were separated using Tukey’s test at *p* ≤ 0.05. Bars represent mean ± SE (*n* = 3). Significant (*p* ≤ 0.05) differences are marked with asterisks. * Significant difference at *p* < 0.05; ** significant difference at *p <* 0.005; *** significant difference at *p* < 0.001; and **** significant difference at *p* < 0.0001.

The accumulations of TC and TA among the four cultivars after three months of salt stress exposure are presented in Figure 7. It is evident that the stress condition contributed to a decrease in TC in all cultivars, with the most pronounced decrease observed in the Mejhoul cultivar (86%) under the 154 mM NaCl condition.

Conversely, all cultivars accumulated higher levels of TA compared with the control condition (Figure 7), with the highest accumulation observed in the Bouskri cultivar (83%) (*p <* 0.0001).

#### 3.2.4. Hydrogen Peroxide Accumulation and Antioxidant Enzyme Activities

**Figure 8:**
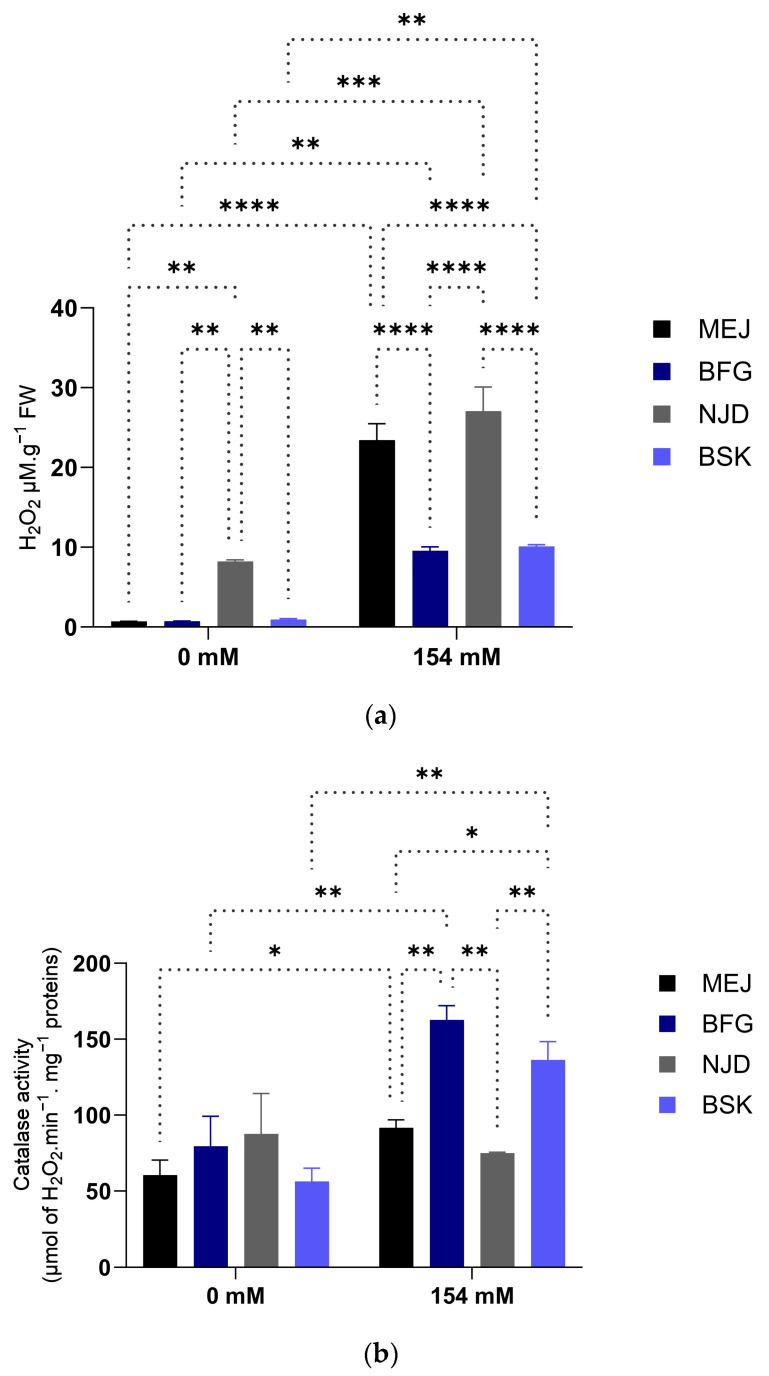

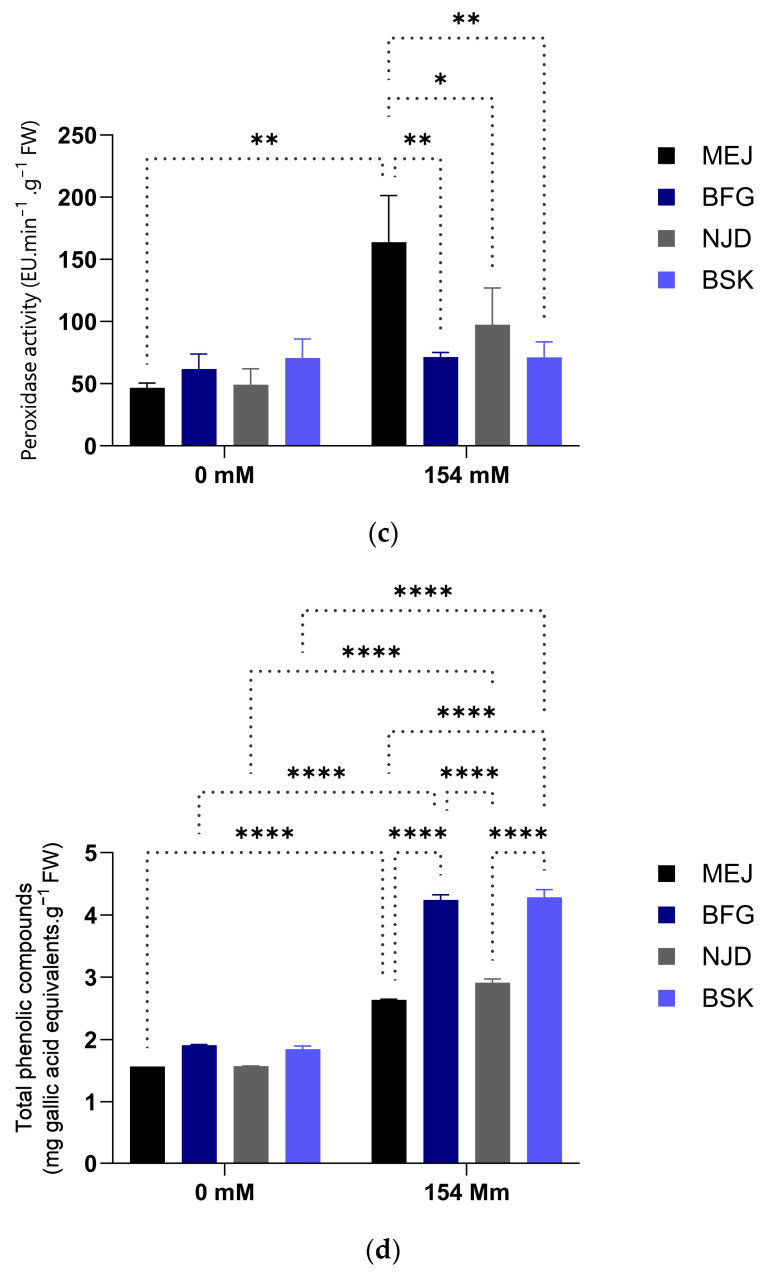
Influence of salt stress on H_2_O_2_ contents, catalase activity, peroxidase activity, and phenolic compounds in the leaves of four Moroccan date palm (*Phoenix dactylifera* L.) cultivars. (**a**) Effect of salt treatment on H_2_O_2_ contents; (**b**) effect of salt treatment on catalase activity; (**c**) effect of salt treatment on peroxidase activity; and (**d**) effect of salt treatment on total phenolic compounds. Means were separated using Tukey’s test at *p* ≤ 0.05. Bars represent mean ± SE (*n* = 3). Significant (*p* ≤ 0.05) differences are marked with asterisks. * Significant difference at *p* < 0.05; ** significant difference at *p* < 0.005; *** significant difference at *p* < 0.001; and **** significant difference at *p* < 0.0001.

The levels of hydrogen peroxide (H_2_O_2_) production in the Boufeggous and Bouskri cultivars exhibited a noticeable upward trend, with concentrations rising from 0.72 µM.g^−1^ FW to 9.54 µM.g^−1^ FW in Boufeggous (representing a 13-fold increase) and from 0.9 µM.g^−1^ FW to 10.08 µM.g^−1^ FW in Bouskri (representing an 11.2-fold increase) (Figure 8). In contrast, the Mejhoul and Nejda cultivars demonstrated higher H_2_O_2_ values, experiencing respective increases of 23.41-fold and 27-fold.

The results also indicate that salt stress induced an increase in peroxidase (POX) and catalase (CAT) activities, as well as an increase in total phenolic contents (Figure 8).

Salt stress had a significant impact on catalase (CAT) activity in the Mejhoul (*p* < 0.05) cultivar, resulting in a 1.5-fold increase compared with the control (0 mM). Similarly, the Boufeggous (2-fold, *p* < 0.005) and Bouskri (2.42-fold, *p* < 0.005) cultivars showed a substantial increase in CAT activity under salt stress conditions. Notably, the Boufeggous cultivar exhibited the highest CAT activity (162.62 μmol of H_2_O_2_.min^−1^.mg^−1^ proteins).

The peroxidase (POX) activity in the Mejhoul cultivar exhibited a significant augmentation under salt stress conditions, with a 3.5-fold increase (*p* < 0.005). In the Nejda cultivar, the POX activity under salt stress showed a 1.9-fold increase compared with the control batch, although this increase was not statistically significant. In the Boufeggous and Bouskri cultivars, the POX activities slightly increased, with 1.1-fold and 1.007-fold increases, respectively, when compared with the control condition. However, these differences were not statistically significant (Figure 8).

Under salt stress conditions (154 mM), all cultivars experienced a significant increase in the accumulation of total phenolic compounds compared with the control (0 mM) (*p* < 0.0001). Particularly, the Bouskri (BSK) and Boufeggous (BFG) cultivars displayed the highest levels of total phenolic compounds under salt stress with 2.32-fold and 2.21-fold increases, respectively (Figure 8). The Mejhoul and Nejda cultivars also exhibited a slight increase in total phenolic compound accumulation compared with the control, with respective 1.6-fold and 1.8-fold increases. Furthermore, significant differences among the cultivars were observed under the 154 mM salt stress condition (*p* < 0.0001).

#### 3.2.5. MFV Values and Genotypes Ranking

The genotypes were assessed for their degree of salinity tolerance using the MFV as a ranking metric, whereby a higher MFV corresponded to a greater level of salinity tolerance, as elucidated by [30]. Detailed evaluation of MFV can be found in Table 3, revealing that Boufeggous and Bouskri exhibited higher mean MFV values, indicative of their enhanced salinity tolerance. Conversely, Mejhoul and Nejda displayed lower mean MFV values, suggesting a relatively lower level of salinity tolerance (Table 3).

Based on these findings, all four genotypes were categorized into two distinct groups: highly tolerant (MFV > 0.5) and sensitive (MFV < 0.3) to salinity. This classification provides valuable insights into the varying degrees of salinity tolerance exhibited by the genotypes under investigation.

#### 3.2.6. Principal Component Analysis

**Figure 9:**
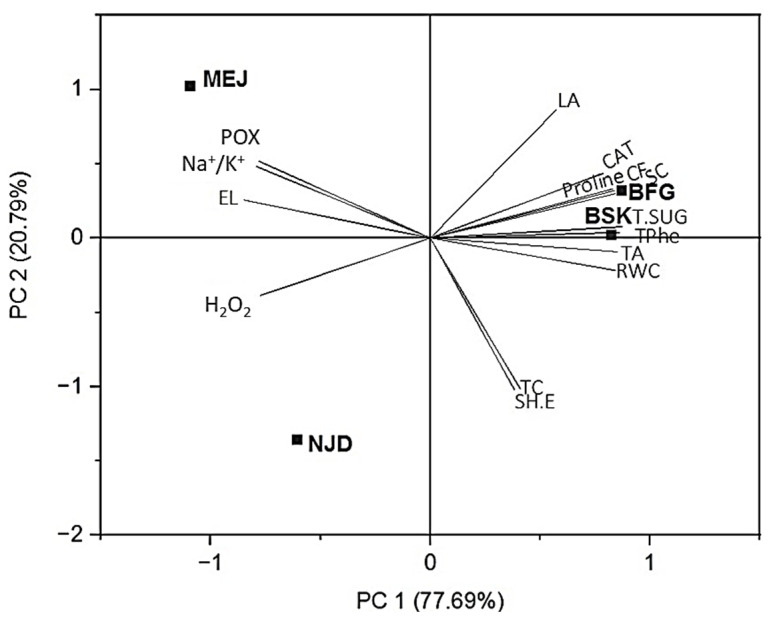
Loading plots of principle components 1 and 2 of the PCA results obtained from morphophysiological and biochemical data of four date palm cultivars subjected to 154 mM NaCl.

The present study provides a comprehensive analysis of the loading plots derived from the principal component analysis (PCA) results obtained from a dataset encompassing morphophysiological and biochemical data of four distinct cultivars of date palm (*Phoenix dactylifera* L.) subjected to a salinity stress of 154 mM NaCl. The loading plots, depicted in Figure 9, offer a visual representation of the distribution patterns of the first two principal components (PC1 and PC2). PC1, accounting for a substantial proportion of the original information (77.69%), emerges as a dominant component in describing the underlying variance within the dataset. PC2, on the other hand, captures a considerable portion of the variance (20.79%). Together, PC1 and PC2 account for an impressive cumulative percentage of 98.48%, signifying their effectiveness in capturing the majority of the dataset’s variability. The utilization of PCA in this study proves instrumental in facilitating the visualization and interpretation of the intricate dataset (Figure 9).

By plotting the morphophysiological and biochemical parameters on the PC1–PC2 space, it becomes evident that these parameters effectively segregate the four cultivars studied. The contributions of the individual parameters to the principal components were explored by examining their loadings on PC1 and PC2. Remarkably, several parameters, including catalase (CAT) activities, leaf area (LA), proline content, chlorophyll fluorescence (CF), stomatal conductance (SC), total sugars (T sugars), and total phenols (T phenols) exhibit positive loadings on the right upper quadrant of the biplot. This clustering implies a positive correlation among these parameters and underscores their collective association with the response of both Boufeggous and Bouskri cultivars. Conversely, sodium/potassium (Na^+^/K^+^) ratio, peroxidase (POX) activities, and electrolyte leakage (EL) manifest as prominent contributors with loadings on the upper-left quadrant of the biplot, primarily associated with the response of the Mejhoul cultivar. Additionally, relative water content (RWC), total chlorophyll (TC), total anthocyanins (TA), and stomatal shoot elongation (Sh.E) are observed in the lower-left portion of the biplot, whereas hydrogen peroxide (H_2_O_2_) shows a lack of correlation with the Nejda cultivar’s response.

## 4. Discussion

The ability of plants to endure and adapt to salt stress is governed by a complex interplay of various physiological, biochemical, and molecular responses [32,33]. Recent research endeavors have been dedicated to unraveling these intricate mechanisms to enhance crop productivity and deepen our understanding of plant reactions to salt stress. Among these mechanisms, osmotic adjustment emerges as a pivotal process characterized by the accumulation of compatible solutes, such as proline, glycine betaine, and sugars. This accumulation plays a crucial role in maintaining cellular water potential and preventing dehydration.

Furthermore, ion homeostasis represents another crucial mechanism involving the regulation of ion uptake and compartmentalization, particularly for sodium (Na^+^) and potassium (K^+^), to counteract the potentially harmful effects of excessive Na^+^ accumulation [33]. Additionally, antioxidants, including ascorbate, glutathione, phenolic compounds, and enzymes such as superoxide dismutase and catalase, assume a vital role in scavenging reactive oxygen species (ROS) generated under salt stress conditions. These antioxidants serve to protect plants from oxidative damage [34].

Date palms exhibit a superior level of adaptation to salt stress in comparison to other tree species. In a previous study [35], a comprehensive screening was conducted on diverse date palm cultivars to evaluate their capacity to withstand salinity. The investigation revealed that specific cultivars demonstrated exceptional resilience to soil salinity levels as high as 12.8 dS m^−1^ without any discernible impact on the phenotype of the seedlings. Additionally, an independent study conducted by another group of researchers [36] demonstrated the ability of different date palm cultivars to endure soil salinity levels of up to 9 dS m^−1^, despite the accumulation of excessive Na^+^ ions in the leaves of plants subjected to high salt concentrations.

In the present study, the Boufeggous and Bouskri cultivars exhibited the lowest Na^+^/K^+^ ratios, whereas the Mejhoul cultivar displayed the highest ratio. These findings align with a previous investigation on date palms by [37], which reported varying Na^+^/K^+^ ratios among date palm cultivars under salt stress. The maintenance of low cytosolic Na^+^/K^+^ ratios, achieved through Na^+^ exclusion, is a critical trait for salt tolerance. Notably, the accumulation of Na^+^ in photosynthetic tissues, particularly in the presence of high concentrations, hinders K^+^ functions, including enzyme deactivation [38,39], turgor maintenance, stomatal regulation, and intracellular pH regulation [40]. It disrupts K^+^ uptake, leading to K^+^ deficiency and the impairment of K^+^-dependent processes.

In this study, all cultivars, except for Mejhoul, displayed increased K^+^ concentrations in the shoots under salt stress. However, due to the higher Na^+^ concentrations in the shoots of the sensitive cultivars, their shoot Na^+^/K^+^ ratios were significantly higher compared with the tolerant cultivars (Figure 5). Previous studies have highlighted the importance of Na^+^/K^+^ ratio as a physiological selection criterion for salt tolerance in various plant species, including tomato [41], chickpea [42], and soybean [43]. The exclusion of Na^+^ from shoots is widely regarded as a fundamental characteristic of salt tolerance in plants [44]. To minimize Na^+^ accumulation in shoots, plants can either restrict its entry from the root symplast to reduce loading, retrieve Na^+^ from the xylem, or export Na^+^ from leaves into the phloem [44].

As for the second mechanism, osmotic adjustment, several studies have reported the accumulation of certain organic osmolytes, such as soluble sugars, proline, and glycine betaine, as a major indicator of cultivar tolerance and an important criterion in screening cultivars for tolerance to abiotic stress [22]. In this study, the BFG and BSK cultivars took the lead in terms of organic osmolytes accumulation, with greatly reduced levels in the other two susceptible cultivars (MEJ and NJD). Such variability in cultivar response to salt stress has been reported in [45,46].

The excessive generation of reactive oxygen species (ROS) is a prevalent outcome observed in response to various stress factors, including salinity. Environmental stressors, such as UV radiation, can induce the production of ROS in plants. To counteract the detrimental effects of these stress conditions, plants activate defensive mechanisms by synthesizing antioxidants, flavonoids, and secondary metabolites. These compounds play a crucial role in protecting the plant by effectively detoxifying ROS and mitigating oxidative damage caused by environmental stress. These protective mechanisms also aid in protecting the plant from drought and contribute to the stabilization of proline and amino acid levels [47]. Ensuring the equilibrium between the generation and degradation of reactive oxygen species (ROS) is imperative for maintaining metabolic functions in challenging circumstances, as any inability to achieve this balance may lead to oxidative damage. Uncontrolled ROS levels can oxidize and inflict damage upon proteins, lipids, and nucleic acids. In the present study, the application of salt stress resulted in varied accumulations among the four cultivars utilized, with the Mejhoul cultivar exhibiting the highest accumulation of H_2_O_2_ in leaves and the Boufeggous cultivar displaying the lowest accumulations. Hence, it was anticipated that exposing date palm vitro plants to NaCl would elevate the levels of antioxidant enzymes and phenols to counteract the oxidative stress induced by salinity, as reported by [48].

Antioxidant enzymes, such as peroxidase (POX) and catalase (CAT), are known to play a crucial role in facilitating plant adaptation to stressful environmental conditions [49]. Our study investigated the impact of salt stress on the activities of POX and CAT in various cultivars of date palm. The findings of our study revealed a consistent increase in the activities of POX and CAT across all date palm cultivars subjected to salt stress. This elevation in enzyme activities corresponded to the enhanced salt tolerance observed, as evidenced by the accumulation of proline and soluble sugars in the Boufeggous and Bouskri cultivars (Figure 6). Notably, the salt-sensitive cultivars exhibited higher levels of POX, whereas the salt-tolerant cultivars displayed higher levels of CAT. These observations align with previous research conducted on rice by [50] reporting an increase in POX activity, specifically in the salt-sensitive cultivars under salt stress. Furthermore, it is well-documented that plants activate potent nonenzymatic antioxidant systems (such as vitamins C and E, carotenoids, flavonoids, and phenolic compounds) in response to stress [51,52]. Consistent with these reports, our study observed significant increases in the levels of these nonenzymatic antioxidants in salt-treated plants. Particularly, a remarkable increase in total phenolic compounds, notably in the BFG and BSK cultivars, was observed, with statistically significant differences from the other two cultivars (*p* < 0.001). At a cellular level, it is well reported that antioxidants play a crucial role in neutralizing reactive oxygen species (ROS) and protecting cells from oxidative damage [53]. In fact, two main types of antioxidants intervene in catalyzing the scavenging of generated ROS in abiotic stresses: enzymatic and nonenzymatic [54]. Enzymatic antioxidants like SOD, CAT, and glutathione peroxidase directly catalyze the breakdown of ROS through enzymatic reactions, serving as primary defenses. Nonenzymatic antioxidants, such as vitamins C and E, carotenoids, and polyphenols, act as secondary defenses by scavenging ROS via nonenzymatic chemical reactions, further shielding cells from damage [55].

In an endeavor to evaluate the impact of salt stress on the chlorophyll content in the leaves of 10 distinct cultivars of date palm, [38] made notable observations regarding the dissimilarities in chlorophyll accumulation observed between the salt-tolerant and salt-sensitive cultivars. In fact, it is widely recognized that photosynthetic traits serve as potent indicators when determining abiotic stress conditions [56]. In our study, the most significant reduction in total chlorophyll content was observed in the salt-treated group of the Mejhoul variety, indicating a substantial impairment of the PSII [57]. This finding is further supported by the results presented in Table 2, as the Mejhoul cultivar exhibited an F_v_/F_m_ ratio of approximately 0.57, which is recognized as falling within the range associated with stress-induced values. Moreover, it is firmly established in the literature that a decline in chlorophyll concentration leads to a reduction in photosynthesis due to diminished light absorption, thereby affecting the conversion of light energy into chemical energy [57]. The plants’ growth and development are thereby influenced, as evidenced by a rudimentary examination of Table 2. Building upon these findings, our investigation revealed that salt stress induced higher levels of anthocyanin production across all date palm cultivars. This aligns with the research conducted by [58] on various rice genotypes, wherein the salt-tolerant cultivars exhibited higher concentrations of anthocyanins com-pared with their salt-sensitive counterparts. Furthermore, our results were consistent with previous studies on eight indica rice genotypes, which demonstrated increased production of anthocyanins under salt stress conditions. The findings of [58] further support these observations by highlighting the antioxidant properties of anthocyanins. It is suggested that anthocyanins act as direct scavengers of reactive oxygen species (ROS) and are believed to mitigate oxidative damage.

From a physiological perspective, the literature extensively documents the impact of salt stress on the photosynthetic apparatus. Numerous studies, including those conducted by [38,59,60], have provided evidence demonstrating alterations in the number and behavior of stomata under salt stress conditions. In the present investigation, the imposition of salinity resulted in a significant reduction in stomatal conductance, which was closely correlated with the observed decrease in the F_v_/f_m_ ratio in both Mejhoul and Nejda plant cultivars (refer to Table 2). Consequently, the growth of these cultivars under salt conditions was impeded, as plant growth is inherently dependent on photosynthesis, which is susceptible to abiotic stress. It is worth noting that one of the primary mechanisms adopted by plants to mitigate water loss is the closure of stomata, which in turn leads to a diminished capacity for CO_2_ uptake and imposes osmotic stress [60,61].

Moreover, a decrease in relative water content (RWC) has the potential to hinder plant growth under salt stress [62]. In our study, RWC declined across all cultivars, with the decline being more pronounced in the susceptible plants, such as Mejhoul, where it dropped below 50.8% (see Figure 4). This finding suggests that RWC constitutes a contributing factor to stress sensitivity in certain susceptible cultivars. Conversely, the effect was less pronounced in the Boufeggous and Bouskri cultivars, as their RWC values were comparable to those reported for the control group. In line with our results, ref. [63] observed a lower level of RWC in tobacco plants subjected to salt stress.

Due to the alterations in biochemical and physiological mechanisms, the phenotypic characteristics of the different genotypes were also impacted, as evidenced by the significant differences observed among cultivars in terms of leaf area, number of neoformed leaves, and nonfunctional leaves, as indicated in Table 2. These findings strongly support the previously described grouping method, which identified Mejhoul as the most sensitive cultivar and Boufeggous as the most tolerant. Notably, leaf area plays a crucial role in influencing photosynthetic performance and the accumulation of photosynthetic products. Consistent with the results presented in Table 2, prior studies have also reported a reduction in leaf area expansion under salinity conditions in wheat [62] and rice [63].

The imposition of 154 mM NaCl concentration as a stressor enabled the categorization of the four employed genotypes into two distinct groups based on their responses to saline conditions. The first group, characterized as salt-sensitive, exhibited a high degree of stability in growth rate and promptly displayed salt-induced chlorosis as a sensing reaction. This category included both the Mejhoul and Najda cultivars. On the other hand, the second group, comprising the Boufeggous and Bouskri cultivars, demonstrated a more pronounced resilience to the stressor, with growth rates surpassing those observed in the control group (e.g., stressed Boufeggous reaching 16 cm compared to 6 cm in the control group and stressed Bouskri reaching 6 cm compared to 1 cm in the control group) and no visible signs of stress. The observed reduction in plant growth under NaCl exposure can be attributed to changes in osmotic pressure resulting from excessive ion accumulation in plants, subsequently leading to nutritional imbalances [64].

Principal component analysis (PCA) is a widely utilized multivariate method employed for classification purposes, enabling the discrimination of samples based on their diverse biological status, origin, or quality. This approach has been extensively documented in the literature [65,66]. In this study, the combination of PCA and membership function value (MFV) was employed to classify the four cultivars into distinct groups, based on various morphophysiological and biochemical parameters, with the aim of discerning their level of salt tolerance. This classification process was accompanied by the utilization of Figure 9 and Table 3, which visually and numerically represented the obtained results.

The PCA successfully distinguished the salt-sensitive cultivars from the other cultivars, thereby validating the findings reported by [66]. Specifically, the PCA effectively separated the salt stress-tolerant cultivars from the sensitive ones, mainly along the PC1 axis, which contained the highest amount of information (77.69%) (Figure 9). Moreover, in accordance with the PCA results, the tolerant cultivars, Boufeggous and Bouskri, exhibited higher proline accumulation, elevated catalase (CAT) activity, increased phenolic compound contents, enhanced stomatal conductance, and elevated chlorophyll fluorescence. Conversely, the sensitive cultivars, Mejhoul and Nejda, displayed higher peroxidase (POX) activities, higher Na^+^/K^+^ ratios, and increased hydrogen peroxide (H_2_O_2_) contents.

In light of the findings presented in this study, there are several perspectives that can be considered for further research and exploration in the field of screening Moroccan date palm cultivars for salt stress tolerance. We recommend delving into the molecular scale to elucidate a comprehensive and well-explained map of the date palm’s response to salt stress in both tolerant and sensitive Moroccan cultivars. By studying gene expression patterns and molecular mechanisms, researchers can gain a deeper understanding of the genetic basis underlying salt stress tolerance in date palms. Additionally, further investigation into the reproductive stage and evaluation of fruit quality and quantity can provide valuable insights into the impact of salt stress on the reproductive performance of Moroccan date palm cultivars. This would help confirm and expand upon the existing studies, providing a more comprehensive understanding of the effects of salt stress on date palm productivity and fruit quality. Furthermore, it is essential to explore additional physiological, phenotypic, and biochemical parameters that may serve as supplementary or alternative indicators for screening salt tolerance in date palms. This could involve investigating other parameters such as antioxidant enzyme activities, osmolyte accumulation, or specific gene expression related to salt stress response.

Considering the impact of climate change on salt stress tolerance in date palm cultivars is an important perspective to explore. Understanding how changing climatic conditions, such as variations in annual rainfall and average temperature, may interact with salt stress can provide insights into the adaptability and resilience of the tolerant date palm cultivars in the face of future environmental challenges. Lastly, it is crucial to consider the development of sustainable agricultural practices that can mitigate the effects of salinity on date palm cultivation. This could involve the use of adapted varieties with broader environmental tolerances, as well as the implementation of irrigation and soil management strategies to minimize the impact of salt stress on crop production.

## 5. Conclusions

The results of this study provide compelling evidence to confidently assert that salt stress induces significant negative physiological, phenotypical, and biochemical changes in date palm (*Phoenix dactylifera* L.), regardless of the cultivar. Furthermore, the response of Mejhoul, Nejda, Boufeggous, and Bouskri varied under the salt stress condition, which triggered different morphophysiological and biochemical responses. Based on these morphophysiological and biochemical traits and relying on the PCA and MFV classifying methods, this study grouped the four cultivars into two distinct groups: one tolerant comprising Boufeggous and Bouskri and one sensitive comprising Nejda and Mejhoul. The sensitive cultivars exhibited distinctive characteristics under salt stress, including a high Na^+^/K^+^ ratio and an increased accumulation of H_2_O_2_. In contrast, the tolerant cultivars demonstrated a greater capacity to accumulate organic osmolytes, such as proline and soluble sugars. These cultivars also displayed a more pronounced antioxidant pattern and maintained a balanced water status, even when exposed to salt stress conditions. Furthermore, the sensitive cultivars demonstrated higher peroxidase (POX) enzyme activity, whereas the tolerant group exhibited higher catalase (CAT) activity.

## Figures and Tables

**Table 1:** Agro-morphological characteristics of the Moroccan date palm genotypes used in this study based on modalities of characteristics according to [21].

Cultivar	Geographical Distribution	Fruit Characteristics		
		Color	Consistency	Maturity
Mejhoul	Tafilalet-Ziz	Dark brown	Half soft	Late
Boufeggous	Moroccan oases	Dark brown	Soft	Season
Nejda	Drâa	Light brown	Half soft	Season
Bouskri	Draa, Bani, Saghro, Todra	Dark brown	Dry	Mid-late

**Table 2:** Morphophysiological data of four date palm (*Phoenix dactylifera* L.) genotypes grown under 154 mM NaCl after three months.

Evaluated Parameter		MEJ	BFG	NJD	BSK
Elongation (cm)	0 mM	5.02 ± 0.15 ^a^	9.23 ± 0.2 ^b^	6.41 ± 0.28 ^c^	4.95 ± 0.83 ^a^
	154 mM	0.70 ± 0.29 ^e^	16.44 ± 0.98 ^k^	0.63 ± 1.47 ^e^	5.71 ± 1.12 ^c^
Leaf area (cm^2^)	0 mM	32.36 ± 1.23 ^a^	27.62 ± 0.41 ^b^	22.1 ± 0.26 ^d^	26.82 ± 1.81 ^b^
	154 mM	17.32 ± 1 ^d^	22.41 ± 0.14 ^b^	8.2 ± 1.21 ^a^	20.13 ± 0.14 ^b^
Electrolyte leakage (%)	0 mM	11.96 ± 0.32 ^ad^	13.11 ± 0.19 ^bd^	12.88 ± 1.51 ^d^	12.06 ± 0.14 ^d^
	154 mM	52.49 ± 1.38 ^a^	21.76 ± 1.29 ^b^	37.98 ± 0.28 ^c^	24.16 ± 0.25 ^d^
Fv/Fm	0 mM	0.7425 ± 0.12 ^a^	0.7625 ± 1.49 ^a^	0.73 ± 0.41 ^a^	0.75 ± 0.21 ^a^
	154 mM	0.57 ± 0.22 ^a^	0.71 ± 0.41 ^b^	0.545 ± 1.32 ^a^	0.72 ± 0.017 ^b^
Stomatal conductance (mmol/m^2^/s)	0 mM	82.64 ± 0.05 ^a^	82.83 ± 0.43 ^a^	86.94 ± 1.83 ^b^	67.95 ± 1.26 ^c^
	154 mM	22.15 ± 1.94 ^a^	42.96 ± 2.22 ^b^	19.35 ± 1.31 ^c^	43.32 ± 1.75 ^e^

Values shown are means (*n* = 40) ± SD; within rows, means followed by different letter are significantly different using Tukey’s test at *p* < 0.05.

**Table 3:** Ranking of date palm genotypes based on MFV. MFV over 0.5 = tolerant (T) and MFV below 0.3 = sensitive (S). Abbreviation: MFV, membership function value.

Genotype	Mean MFV	Ranking
MEJ	0.26	S
BFG	0.71	T
NJD	0.27	S
BSK	0.82	T

## Data Availability

The datasets generated during and/or analyzed during the current study are available from the corresponding author on reasonable request.

## Abbreviations

The following abbreviations are used in this manuscript:

**ANOVA**	Analysis of variance
**BFG**	Boufeggous
**BSK**	Bouskri
**CAT**	Catalase
**CCI**	Chlorophyll content index
**CF**	Chlorophyll fluorescence
**EC**	Electrical conductivity
**EDTA**	Ethylenediaminetetraacetic acid
**EL**	Electrolyte leakage
**H_2_O_2_**	Hydrogen peroxide
**HCl**	Hydrochloric acid
**HNO_3_**	Nitric acid
**LA**	Leaf area
**MEJ**	Mejhoul
**MFV**	Membership function value
**NaCl**	Sodium chloride
**NJD**	Nejda
**PCA**	Principal component analysis
**POX**	Peroxidases
**PSII**	Photosystem II
**PVPP**	Polyvinylpolypyrrolidone
**ROS**	Reactive oxygen species
**RWC**	Relative water content
**SC**	Stomatal conductance
**SH. E**	Shoot elongation
**STC**	Salt tolerance coefficient
**T.SUG**	Total soluble sugars
**TA**	Total anthocyanin
**TC**	Total chlorophyll
**TCA**	Trichloroacetic acid
**TPHe**	Total phenolic compounds
